# Impact of a Practice-wide Switch from Traditional Right Ventricular Pacing to Left Bundle Branch Area Pacing

**DOI:** 10.19102/icrm.2025.16064

**Published:** 2025-06-15

**Authors:** David Fritz, Ben Ose, Hannah Zerr, Maci Clark, Caroline Trupp, Amulya Gupta, Ahmed Shahab, Seth H. Sheldon, Amit Noheria

**Affiliations:** 1 The University of Kansas School of Medicine, Kansas City, KS, USA; 2 Department of Cardiovascular Medicine, The University of Kansas Medical Center, Kansas City, KS, USA; 3 Department of Cardiology, Southern Illinois University School of Medicine, Springfield, IL, USA

**Keywords:** Conduction system pacing, left bundle branch area pacing, pacemaker, pacing-induced cardiomyopathy

## Abstract

Left bundle branch area pacing (LBBAP) may mitigate pacing-induced cardiomyopathy (PICM) and is increasingly favored over traditional right ventricular pacing (RVP). We sought to evaluate the impact of a practice-wide switch from RVP to LBBAP. We switched practice from RVP to primarily LBBAP at our center in 2020. A retrospective review was conducted to compare patients who underwent LBBAP from 2020–2023 with controls who underwent RVP from 2018–2019. The LBBAP (n = 288; age, 73.3 ± 10.7 years; left ventricular ejection fraction [LVEF], 56.9% ± 11.4%) and RVP (n = 172) groups were similar in terms of age, body mass index, hypertension, diabetes, and LVEF. The LBBAP group as compared to the RVP group had fewer women (38% vs. 51%; *P* = .006) and longer intrinsic conducted QRS durations (117 ± 28 vs. 110 ± 30 ms; *P* = .04). LBBAP devices required longer implant (102 vs. 67 min) and fluoroscopy (9.3 vs. 6.9 min) times but resulted in shorter paced QRS durations (122 ± 20 vs. 145 ± 24 ms; all *P* < .0001). At 3 months, LBBAP patients had higher sensing (13.8 ± 6.1 vs. 12.0 ± 5.6 mV; *P* = .007), lower pacing impedance (543 ± 98 vs. 576 ± 150 Ω; *P* = .008), and similar capture threshold (0.78 ± 0.24 vs. 0.76 ± 0.35 V; *P* = .5) values. Device-related adverse events were similar between the groups (LBBAP 8.7% vs. RVP 8.8%; *P* = 1.0), which included ventricular lead dislodgement (2.1% vs. 0.6%; *P* = .3). There were no differences in hazard rates of all-cause mortality (*P* = .5) or heart failure (HF) hospitalizations (*P* = .07). In a subgroup of patients with ≥20% ventricular pacing, the average LVEF change during follow-up in the LBBAP group as opposed to the RVP group was +1.6% ± 12.9% versus −3.8% ± 12.0% (*P* = .03), the average left ventricular internal diameter at end-diastole change was −0.18 ± 0.73 cm versus +0.16 ± 0.45 cm (*P* = .006), and there were no differences in the hazard rate of all-cause mortality (*P* = .6) or HF hospitalizations (*P* = 1.0). Our results suggest there were no adverse consequences of the practice-wide switch from RVP to LBBAP. LBBAP was associated with longer procedure and fluoroscopy times but resulted in narrower paced QRS durations and less PICM.

## Introduction

A pacemaker is an effective treatment for cardiac conduction disease and is often implanted for irreversible symptomatic bradycardia or pathologic atrioventricular block. Traditionally, ventricular pacing has been accomplished by myocardial capture from a pacing lead placed in the right ventricular (RV) endocardium without targeting the conduction system. However, single-site RV pacing (RVP) can cause dyssynchronous left ventricular (LV) activation, leading to an increased risk for heart failure (HF).^1,2^ Consequently, a drop in the LV ejection fraction (LVEF) of <50% (pacing-induced cardiomyopathy [PICM]) occurs in 10%–20% of patients receiving chronic RVP.^3,4^

Different pacing strategies have been proposed to mitigate or treat PICM. These include biventricular pacing (BiVP) and conduction system pacing. BiVP uses an additional lead, usually in an epicardial LV vein, for dual-site pacing across the LV to decrease the ventricular dyssynchrony. However, BiVP implantation is a longer, more complex procedure with challenges and complications that include coronary sinus dissection, high capture thresholds, phrenic nerve stimulation, suboptimal lead location, lead dislodgement, non-physiological electrical activation from the epicardium, and the need for an extra pacing lead.^5^ Consequently, BiVP is routinely offered only to patients with pre-existing LV dysfunction.^6^

Conduction system pacing started out as His-bundle pacing (HBP) but is now implemented primarily with left bundle branch area pacing (LBBAP).^7^ HBP attempts to recruit the bundle branches at the level of the atrioventricular bundle of His and leverage the intrinsic Purkinje network to restore synchronous ventricular activation via a narrow normal QRS complex.^8^ While HBP may be physiologically superior to RVP and BiVP, the procedure itself is technically difficult. The His bundle is difficult to recruit and may be unsuccessful in 10%–20% of cases.^9^ Further, the long-term stability of HBP leads can be unpredictable, and concerns remain about the future development of bundle branch blocks below the level of His bundle capture.

LBBAP mitigates such issues by using a lead implanted from the right and penetrated into the interventricular septum to stimulate the left bundle branches. By directly or indirectly recruiting the native LV electrical system, it enables the spread of depolarization synchronously throughout the LV and the QRS width decreases. This is putatively a superior strategy for patients undergoing permanent ventricular pacing.^10^ LBBAP has been shown to reverse PICM.^11^ LBBAP, in comparison to BiVP, has demonstrated shorter QRS complex durations, better intraventricular synchrony, and a modest hemodynamic improvement.^12,13^ Further, as compared to HBP, LBBAP results in lower pacing thresholds, higher R-wave amplitudes, shorter procedure times, and greater implant success.^14–16^

Despite widespread clinical adoption, there is an absence of comparative randomized clinical trial data on the short- and long-term outcomes of LBBAP as compared to RVP. Not enough is known about the impact of switching routine practice from RVP to LBBAP in a real-world setting. We evaluated the outcomes of a practice-wide shift from conventional RVP to LBBAP at our center.

## Methods

Our clinical practice of permanent pacemaker implants at a single large tertiary care center, The University of Kansas Medical Center, Kansas City, changed from primarily RVP to primarily LBBAP in 2020. A retrospective chart review was done to assess patients ≥18 years of age who underwent an LBBAP lead implantation between 2020 and 2023 compared to controls who underwent RVP from 2018–2019. The control patients were collected as part of a different retrospective study comparing venous access techniques for cardiac implantable electronic device implantation.^17^

Baseline patient and procedural characteristics were retrospectively recorded. Implants were classified as either de novo implants or upgrades/revisions (with or without lead extraction). A device-related adverse event was defined as implant-related deep venous thrombosis, infection at the site of implantation, any lead dislodgement, any lead failure, any lead perforation, pocket hematoma requiring evacuation or interruption of anticoagulation, pneumothorax, or pericardial effusion/tamponade requiring pericardiocentesis. Post-procedural follow-up data from multiple electrocardiograms (ECGs), device interrogations, and echocardiograms were recorded. As per standard practice, patients undergoing LBBAP received a 12-lead ECG the morning after device implant to record the paced QRS morphology. We compared the paced QRS duration and LV activation time (LVAT) between LBBAP patients and RVP controls. Data from device interrogations were used to compare LBBAP electrical parameters, such as sensing, pacing impedance, and capture thresholds, to those in RVP controls. Echocardiography was used to determine LVEF, LV internal diameter at end-diastole (LVIDd), LV internal diameter at end-systole, LV end-diastolic volume, and LV end-systolic volume at the time of implant and during follow-up.

Subgroup analyses were performed for patients with ≥20% ventricular pacing burden documented on either 3- or 12-month post-implant device interrogation reports.

Patient data were retrospectively entered and stored securely in an electronic database (Research Electronic Data Capture [REDCap]). Analysis was conducted using JMP Pro version 15 (SAS Institute, Cary, NC, USA). Continuous variables were expressed as mean ± standard deviation values. Categorical variables were expressed as frequency and percentages. Student’s *t* test and Fisher’s exact test were used to compare continuous and categorical values, respectively. We considered a two-tailed *P* value of ≤.05 to indicate statistical significance.

## Results

### Baseline characteristics

There were 288 patients who underwent LBBAP (73.3 ± 10.7 years) between 2020 and 2023 performed by seven operators. These patients were compared to 172 RVP controls (71.8 ± 11.4 years) who underwent implantation between 2018 and 2019, also by seven operators. Baseline patient characteristics can be seen in **Table 1**. There were no significant differences in age or body mass index, but fewer women underwent LBBAP (38% vs. 51%; *P* = .006). Patients who underwent LBBAP had longer average intrinsic QRS durations compared to RVP controls (117 ± 28 vs. 110 ± 30 ms; *P* = .04). There was no difference in baseline medical comorbidities, including hypertension, diabetes, chronic obstructive pulmonary disease, obstructive sleep apnea, and coronary artery disease. There were no statistical differences in medical therapy between the two groups except for the higher use of sodium–glucose cotransporter 2 inhibitors (*P* = .003) among LBBAP (7.3%) versus RVP (1.2%) patients, as shown in **Supplementary Table 1**. Patients in both groups had similar baseline LVEFs (56.9% ± 11.4% vs. 56.3% ± 9.8%; *P* = .7).

**Table 1:** Comparison of Baseline Characteristics Between LBBAP and Traditional RVP

Variable	LBBAP (n = 288)	RVP (n = 172)	*P*
Implant years	2020–2023	2018–2019^a^	
Duration of follow-up, months	17.1 ± 6.4	59.2 ± 8.9	<.0001*
Age, years	73.3 ± 10.7	71.8 ± 11.4	.1
Female sex	108 (38%)	87 (51%)	.006*
White race	238 (85%)	139 (84%)	.5
BMI, kg/m^2^	29.9 ± 6.7	29.7 ± 6.6	.8
Hypertension	227 (79%)	140 (81%)	.5
Diabetes mellitus	98 (34%)	52 (30%)	.4
Chronic obstructive pulmonary disease	47 (16%)	22 (13%)	.3
Obstructive sleep apnea	87 (30%)	51 (32%)	.7
Coronary artery disease	139 (48%)	81 (47%)	.8
Intrinsic conducted QRS duration, ms^b^	117 ± 28	110 ± 30	.04*
LV ejection fraction, %^c^	56.9 ± 11.4	56.3 ± 9.8	.7

*Abbreviations:* BMI, body mass index; LBBAP, left bundle branch area pacing; LV, left ventricular; RVP, right ventricular pacing. ^a^The control group includes six patients who received RV pacing leads during 2020–2023. ^b^n = 156 (54%) and n = 120 (70%), respectively, for cases and controls. ^c^n = 252 (88%) and n = 131 (76%), respectively, for cases and controls. *Statistically significant.

**Supplementary Table 1:** Distribution of Medical Therapy Between the Two Groups

Medication Class	LBBAP (n = 288)		RVP (n = 172)		*P*
	n	%	n	%	
β-Blocker	129	44.8%	71	41.3%	.5
ACE inhibitor/ARB	113	39.2%	56	32.6%	.2
Neprilysin inhibitor–ARB	10	3.5%	2	1.2%	.2
Aldosterone antagonist	42	14.6%	17	9.9%	.2
SGLT2 inhibitor	21	7.3%	2	1.2%	.003*
Loop/thiazide diuretic	110	38.2%	54	31.4%	.2
Anticoagulant	120	41.7%	84	48.8%	.2

*Abbreviations:* ACE, angiotensin-converting enzyme; ARB, angiotensin receptor blocker; LBBAP, left bundle branch area pacing; RVP, right ventricular pacing; SGLT2, sodium–glucose cotransporter 2. *Statistically significant.

### Procedural characteristics

These results are shown in **Table 2**. Both groups received predominantly de novo device implants instead of upgrades or revisions, though there were fewer de novo implants performed in the LBBAP group compared to the RVP group (91% vs. 98%; *P* = .0008). The RVP lead location in the control group was classified by the implanting operator as apical in 9 (5.2%) and as non-apical in the remaining 163 (94.8%) patients. The models of the leads used for the LBBAP and RVP groups are provided in **Supplementary Table 2**. There were similar numbers of implantable cardioverter-defibrillators (7% vs. 3%; *P* = .06) but more cardiac resynchronization therapy generators (9% vs. 2%; *P* = .001) implanted in the LBBAP group compared to the RVP group. Among de novo single- or dual-chamber device implantations, procedure durations (102 ± 37 vs. 67 ± 30 min; *P* < .0001) and fluoroscopy times (9.3 ± 8.7 vs. 6.9 ± 8.1 min; *P* < .0001) were longer in the LBBAP group than in the RVP group.

**Table 2:** Comparison of Procedural Variables Between LBBAP and Traditional RVP

Variable	LBBAP (n = 288)	RVP (n = 172)	*P*
New implant (not an upgrade/revision)	261 (91%)	169 (98%)	.0008*
Implantable cardioverter-defibrillator	21 (7%)	5 (3%)	.06
Cardiac resynchronization therapy generator	26 (9%)^a^	3 (2%)	.001*
Implant duration, min^b^	102 ± 37	67 ± 30	<.0001*,^c^
Fluoroscopy time, min^b^	9.3 ± 8.7	6.9 ± 8.1	<.0001*,^c^
Bipolar paced QRS duration, ms^d^	121.5 ± 20.0	144.9 ± 23.7	<.0001*
LV activation time, ms^e^	73.8 ± 20.1	88.0 ± 23.7	.002*
V6–V1 interpeak time, ms	36.0 ± 16.2	N/A	**—**

*Abbreviations:* CRT, cardiac resynchronization therapy; ICD, implantable cardioverter-defibrillator; LBBAP, left bundle branch area pacing; LV, left ventricular; N/A, not available; RV, right ventricular; RVP, right ventricular pacing. ^a^Of these 26 patients, 17 had LBBAP + RV/ICD leads, 6 had failed LBBAP and instead received biventricular pacing with RV + coronary sinus leads, and 3 had LBBAP-optimized CRT with LBBAP + coronary sinus leads. ^b^New single- or dual-chamber implantation only. ^c^Non-parametric Kruskal–Wallis test. ^d^Available in 246 (85%) and 76 (44%) patients, respectively. ^e^Available in 236 (82%) and 34 (18%) patients, respectively. *Statistically significant.

**Supplementary Table 2:** Distribution of Lead Models in the LBBAP and RVP Groups

Lead Model	LBBAP		RVP	
	n	%	n	%
SelectSecure 3830^a^	255	91.4%	2	1.2%
5076^a^	0	0.0%	88	51.2%
Ingevity^b^	20	7.2%	28	16.3%
FineLine^b^	0	0.0%	16	9.3%
Solia^c^	2	0.7%	31	18.0%
Tendril^d^	0	0.0%	6	3.5%
Other	2	0.7%	1	0.6%

*Abbreviations:* LBBAP, left bundle branch area pacing; RVP, right ventricular pacing. ^a^Medtronic (Minneapolis, MN, USA). ^b^Boston Scientific (Marlborough, MA, USA). ^c^Biotronik (Berlin, Germany). ^d^Abbott (Chicago, IL, USA).

### Electrocardiogram result and electrical function

**Figure 1:**
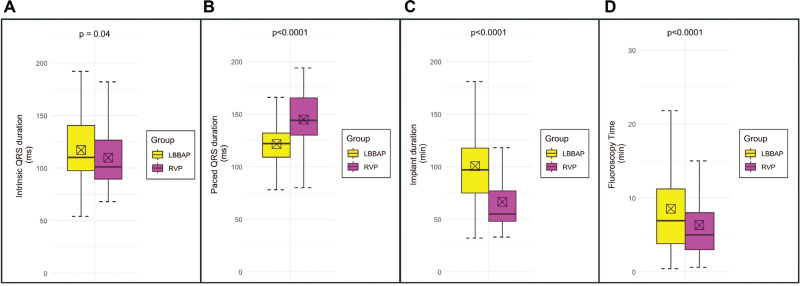
**(A)** Intrinsic QRS duration, **(B)** paced QRS duration, **(C)** implant procedure duration, and **(D)** fluoroscopy time for the entire study population (n = 460). *Abbreviations:* LBBAP, left bundle branch area pacing; RVP, right ventricular pacing.

Ventricular paced ECG data are shown in **Table 2**. Day 1 post-implant ECGs documenting the ventricular paced QRS duration were available for review in 246 (85%) and 76 (44%) patients, respectively, in the two groups. LVAT was able to be measured (presence of the R-wave in lead V6) in 236 (82%) and 34 (18%) patients. When available, the LBBAP leads as compared to the RVP controls were associated with shorter paced QRS durations (121.5 ± 20.0 vs. 144.9 ± 23.7 ms; *P* < .0001) and LVATs (73.8 ± 20.1 vs. 88.0 ± 23.7 ms; *P* = .002). **Table 3** shows the electrical parameters of the pacing leads in the two groups. On day 1 post-implant, LBBAP leads had similar sensing (12.3 ± 5.7 vs. 11.3 ± 5.7 mV, *P* = .1) and pacing impedance (625 ± 106 vs. 629 ± 127 Ω, *P* = .7) values but lower capture thresholds (0.47 ± 0.19 vs. 0.55 ± 0.23 V; *P* = .0004) compared to RVP leads; however, the difference in capture threshold disappeared at 3-month follow-up (0.78 ± 0.24 vs. 0.76 ± 0.35 V; *P* = .5). Additionally, at 3 months, the sensing was higher with LBBAP than with RVP (13.8 ± 6.1 vs. 12.0 ± 5.6 mV; *P* = .007) and the pacing impedance was lower (543 ± 98 vs. 576 ± 150 Ω; *P* = .008). The results of procedural characteristics and ECG are summarized in **Figure 1**.

**Table 3:** Comparison of Lead Electrical Parameters Between LBBAP and Traditional RVP

Variable	LBBAP (n = 288)	RVP (n = 172)	*P* ^a^
1 day post-implant^b^			
Sensing, mV	12.3 ± 5.7	11.3 ± 5.7	.1
Pacing impedance, Ω	625 ± 106	629 ± 127	.7
Capture threshold, V	0.47 ± 0.19	0.55 ± 0.23	.0004*
3 months post-implant^b^			
Sensing, mV	13.8 ± 6.1	12.0 ± 5.6	.007*
Pacing impedance, Ω	543 ± 98	576 ± 150	.008*
Capture threshold, V	0.78 ± 0.24	0.76 ± 0.35	.5

*Abbreviations:* LBBAP, left bundle branch area pacing; RVP, right ventricular pacing. ^a^Independent-samples *t* test. ^b^Bipolar configuration. *Statistically significant.

### Device-related adverse events

**Figure 2:**
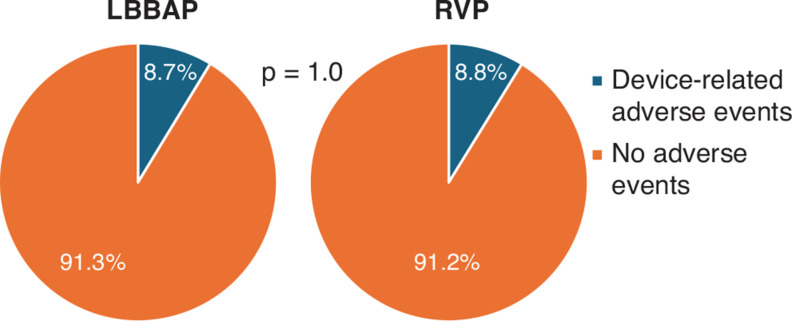
Summary of overall device-related adverse events for the entire study population (N = 460). *Abbreviations:* LBBAP, left bundle branch area pacing; RVP, right ventricular pacing.

Adverse events related to the devices are summarized in **Table 4** and **Figure 2**. The overall device-related adverse event rate was similar between the LBBAP and RVP (8.7% vs. 8.8%; *P* = 1.0) groups. The most common adverse event was any lead dislodgement, with 10 (3.5%) dislodgements observed in the LBBAP group and 4 (2.3%) observed in the RVP group. In the LBBAP subset, 6 out of 10 dislodgements involved the LBBAP lead and 4 involved the right atrial lead. In the RVP subset, one out of four involved the RVP lead and three involved the right atrial lead. There were no statistical differences in any specific device-related adverse event or 30-day outcomes between the two groups.

**Table 4:** Comparison of Device-related Adverse Events and Clinical Outcomes Between LBBAP and Traditional RVP.

Variable	LBBAP (n = 288)	RVP (n = 172)	*P*
Implant years	2020–2023	2018–2019	
Total device-related adverse events	25 (8.7%)	15 (8.8%)	1.0
Procedure-related complications (≤30 days)	13 (4.5%)	8 (4.7%)	1.0
Implant-related DVT	1 (0.3%)	2 (1.2%)	0.6
Device infection	1 (0.3%)	1 (0.6%)	1.0
Any lead perforation needing revision	2 (0.7%)^a^	0 (0%)	0.5
Pericardial effusion requiring intervention	3 (1.1%)	3 (1.7%)	0.7
Pneumothorax	4 (1.4%)	0 (0%)	0.3
Pocket hematoma^b^	2 (0.7%)	2 (1.2%)	0.6
Lead-related adverse events	12 (4.2%)	7 (4.1%)	1.0
Atrial lead dislodgement	4 (1.4%)	3 (1.7%)	0.7
Ventricular lead dislodgement^c^	6 (2.1%)	1 (0.6%)	0.3
Atrial lead failure	1 (0.3%)	0 (0%)	1.0
Ventricular lead failure	1 (0.3%)	3 (1.7%)	0.1
30-day outcomes	2 (0.7%)	3 (1.7%)	0.4
Myocardial infarction	0 (0%)	0 (0%)	1.0
Stroke/TIA	0 (0%)	2 (1.2%)	0.1
All-cause death^d^	2 (0.7%)	1 (0.6%)	1.0
1-year mortality	15 (5%)	10 (6%)	0.8
Outcomes during entire follow-up			
Duration of follow-up, months	17.1 ± 6.4	59.2 ± 8.9	<0.0001*
All-cause mortality during follow-up	25 (8.7%)	49 (28.5%)	<0.0001*
Overall HF hospitalization	24 (8.3%)	19 (11.1%)	0.4
Days in hospital for HF	1.0 ± 5.5	1.4 ± 6.2	0.3^e^
HF hospital days/year	0.7 ± 3.6	0.3 ± 1.5	0.4^e^

*Abbreviations:* DVT, deep venous thrombosis; HF, heart failure; LBBAP, left bundle branch area pacing; RVP, right ventricular pacing; TIA, transient ischemic attack. ^a^One atrial and one ventricular lead. ^b^No intervention required. ^c^Post-procedural. ^d^None of the deaths were due to implant-related causes. ^e^Non-parametric Kruskal–Wallis test. *Statistically significant.

### Long-term outcomes

Both groups were followed up to evaluate long-term outcomes **(Table 4)**. The LBBAP group had a shorter follow-up period than the RVP group (17.1 ± 6.4 vs. 59.2 ± 8.9 months; *P* < .0001). During this shorter follow-up, there were fewer all-cause deaths noted in the LBBAP group, with 25 (8.7%) recorded versus 49 (28.5%) in the RVP group. There was no difference in the number of HF hospitalizations, with 24 (8.3%) versus 19 (11.1%) respectively noted in the LBBAP and RVP groups. Time-to-event Cox proportional hazard ratios of clinical events during follow-up showed no significant difference in the hazard rate of all-cause mortality or HF hospitalizations between the two groups **(Table 5)**.

**Table 5:** Hazard Ratios of Clinical Events Between LBBAP and Traditional RVP During Follow-up

Endpoint	Entire Study Population (N = 460)			Patients with ≥20% Ventricular Pacing (n = 209)		
	HR	95% CI	*P*	HR	95% CI	*P*
Unadjusted models						
All-cause mortality	1.21	0.66–2.23	.5	0.81	0.35–1.88	.6
HF hospitalization	1.93	0.93–4.04	.07	1.02	0.38–2.73	1.0
Models adjusted for age, sex, baseline LV ejection fraction, and baseline intrinsic QRS duration						
All-cause mortality	1.14	0.62–2.11	.7	0.87	0.37–2.06	.8
HF hospitalization	1.79	0.86–3.76	.1	1.09	0.40–3.00	.9

*Abbreviations:* CI, confidence interval; HR, hazard ratio; HF, heart failure; LBBAP, left bundle branch area pacing; LV, left ventricular; RVP, right ventricular pacing.

### Subgroup analysis of patients with ≥20% ventricular pacing

**Figure 3:**
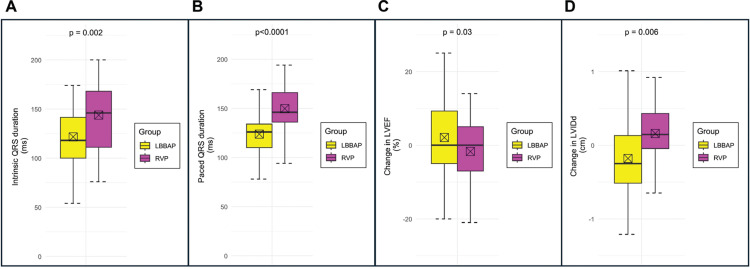
**(A)** Intrinsic QRS duration, **(B)** paced QRS duration, **(C)** change in LVEF, and **(D)** change in LVIDd for patients with ≥20% ventricular pacing (n = 209) during follow-up. *Abbreviations:* LBBAP, left bundle branch area pacing; LVEF, left ventricular ejection fraction; LVIDd, left ventricular internal dimension at end-diastole; RVP, right ventricular pacing.

We evaluated a subgroup of patients with ventricular pacing >20% at either 3-month or 12-month follow-up to evaluate patients at greater risk for PICM **(Table 6** and **Figure 3)**. This subgroup included 151 (52%) LBBAP cases and 58 (34%) RVP controls. There were no significant differences in the baseline clinical or echocardiographic characteristics, while baseline ECG measurements revealed a shorter intrinsic conducted QRS duration (121.2 ± 27.2 vs. 143.5 ± 34.1 ms; *P* = .002) and shorter paced QRS duration (123.8 ± 20.9 vs. 149.7 ± 21.5 ms; *P* < .0001) in the LBBAP group compared to RVP controls.

**Table 6:** Comparison of LBBAP and Traditional RVP in the Subgroup of Patients with ≥20% Ventricular Pacing During Follow-up (at 3- or 12-Month Interrogation)^a^

Variable	LBBAP n = 151 (52%)	RVP n = 58 (34%)	*P*
Baseline clinical characteristics			
Age, years	74.3 ± 10.4	74.8 ± 10.1	.76
Female	53 (35%)	29 (50%)	.058
White race	127/147 (86%)	50/57 (88%)	.80
BMI, kg/m^2^	29.9 ± 6.7	30.5 ± 6.8	.49
Hypertension	123 (81%)	50 (86%)	.54
Diabetes mellitus	47 (31%)	19 (33%)	.87
Chronic obstructive pulmonary disease	25 (17%)	11 (19%)	.69
Obstructive sleep apnea	40 (26%)	18 (33%)	.38
Coronary artery disease	75 (50%)	34 (59%)	.28
Electrocardiogram			
Intrinsic conducted QRS duration, ms	121.2 ± 27.2	143.5 ± 34.1	.002*
Bipolar paced QRS duration	123.8 ± 20.9	149.7 ± 21.5	<.0001*
Ventricular pacing burden, %^b^	98.8 (84.0–99.8)	90.0 (43.0–99.3)	.01*,^c^
Baseline echocardiogram*			
LV ejection fraction, %	56.0 ± 11.2	56.0 ± 9.8	.96
LV internal dimension at end-diastole, cm	4.7 ± 0.7	4.5 ± 0.1	.24
LV internal dimension at end-systole, cm	3.3 ± 0.7	3.2 ± 0.7	.25
LV end-diastolic volume, mL	125.8 ± 46.5	108.9 ± 47.5	.43
LV end-systolic volume, mL	55.6 ± 33.0	45.6 ± 23.5	.36
Follow-up			
Duration of follow-up, months	16.0 ± 6.4	45.1 ± 19.6	<.0001*
Change in LVEF, %^d^	1.6 ± 12.9	−3.8 ± 12.0	.03*
Change in LVIDd, cm^d^	−0.18 ± 0.73	0.16 ± 0.45	.006*
1-year all-cause mortality	8 (5.3%)	4 (6.9%)	.7
Overall all-cause mortality	13 (9.6%)	27 (46.6%)	<.0001*
Overall HF hospitalization	13 (8.6%)	11 (19.0%)	.05*
Days in hospital for HF	1.0 ± 5.3	1.5 ± 4.5	.04*
HF hospital days/year	0.8 ± 3.8	0.3 ± 0.9	.06

*Abbreviations:* BMI, body mass index; HF, heart failure; IQR, interquartile range; LBBAP, left bundle branch area pacing; LV, left ventricular; LVEF, left ventricular ejection fraction; LVIDd, left ventricular internal diameter at end-diastole; RVP, right ventricular pacing. ^a^From available data. ^b^Reported as median (IQR) from 12-month interrogation. ^c^Non-parametric (Kruskal–Wallis) test. ^d^Last available LVEF/LVIDd from 3-, 12-, or 24-month echocardiogram referenced to baseline LVEF/LVIDd.

The follow-up duration in patients with ≥20% ventricular pacing was shorter for LBBAP (16.0 ± 6.4 vs. 45.1 ± 19.6 months; *P* < .0001). The change in LVEF (minimum LVEF of 3-, 12-, 24-, and 36-month follow-up referenced to baseline LVEF) in the LBBAP group was +1.6% ± 12.9% compared to that in the RVP group (−3.8% ± 12.0%; *P* = .03). Similarly, LBBAP was also associated with LVIDd change by −0.18 ± 0.73 cm versus +0.16 ± 0.45 cm, respectively (*P* = .006). Overall, during the differential follow-up durations, LBBAP patients had lower rates of all-cause mortality (9.6% vs. 46.6%, *P* < .0001) and HF hospitalization (8.6% vs. 19.0%; *P* = .05), but there were no statistical differences in time-to-event proportional hazards models.

## Discussion

LBBAP is an attractive alternative to RVP with increased adoption in clinical practice. Ours is one of the first studies describing the experience of a practice-wide shift from RVP to LBBAP.

### Key findings

First, LBBAP was associated with lower paced QRS durations, shorter LVATs, lower pacing impedance, and higher sensing compared to RVP. Second, LBBAP was not associated with a decline in LVEF as was the case with RVP. Third, LBBAP implantation resulted in longer total implant duration and fluoroscopy times compared to RVP. Finally, despite higher complexity, LBBAP was not associated with a higher rate of device- or lead-related adverse events.

### Baseline differences

There were fewer upgrades/revisions in the RVP controls (2%) compared to the LBBAP group (9%), partly due to the systematic exclusion of patients with pre-existing device leads from the RVP controls who were collected for an unrelated prior research project on ultrasound-guided axillary vein access.^17^ Although the treated population in both groups was relatively homogenous, there were two key differences. In the LBBAP group, there were fewer women (38% vs. 51%), and the intrinsic conducted QRS duration was also slightly longer (117 ± 28 vs. 110 ± 30 ms). The electrical properties of the heart vary between men and women, potentially influencing the interpretation of our results. For instance, previous studies have indicated that women typically have shorter QRS durations and higher atrial thresholds compared to men.^18,19^ Additionally, research on leadless pacemakers has demonstrated that women exhibit higher impedance than men.^20^ However, these differences are generally minor, with various studies presenting conflicting findings, and are likely to have had only a minimal impact on the observed outcomes.

### Procedural characteristics

Several procedural variables differed between the two groups. In the LBBAP group, a greater proportion of implants were upgrades/revisions rather than new de novo implants compared to those in the RVP group (9% vs. 2% new implants). Previous studies have shown no difference in clinical response between upgrades and de novo implantation, suggesting that the uneven distribution of implantation types has minimal impact on the interpretation of our study.^21^

As anticipated, the paced QRS duration was much shorter in the LBBAP group compared to RVP controls, indicating the recruitment of the native conduction system. Further, average fluoroscopy times and implant durations were longer in LBBAP patients compared to RVP controls, which is replicated in previous studies and is reflective of the more complex nature of LBBAP implantation.^22^ Extended fluoroscopy times during procedures introduce risks associated with higher radiation doses for both operators and patients. Operators can potentially mitigate this risk by reducing the fluoroscopy frame rate, using ultrasound-guided axillary venous access, or employing techniques such as echocardiography or electroanatomic mapping when suitable.^23,24^

### Device-related adverse events and outcomes

No statistically significant differences were found in device-related adverse events, 30-day outcomes, 1-year mortality, and HF hospitalizations between the LBBAP and RVP cohorts. In contrast, a 2023 meta-analysis comparing LBBAP with RVP revealed that, while total lead-related complications were similar in both groups, the LBBAP group exhibited lower all-cause mortality and a reduced risk of HF hospitalization over a 16-month follow-up period.^25^ In our analysis, the hazard rates of HF hospitalization or death were not different in the two groups.

### Electrical parameters

Compared to RVP, the capture threshold was slightly lower in the LBBAP group on day 1 post-implant, although the difference vanished at 3 months post-implant. A meta-analysis pooling data from 25 studies revealed no discernible difference in the capture threshold between the two groups immediately post-procedure, suggesting that the small disparity observed in our data may be a random artifact rather than a substantive trend.^25^ Further, consistent with the findings from previous studies, at 3 months post-implant, the LBBAP group had higher sensing and lower impedance than the RVP group, which can be attributed to the placement of leads in the muscular interventricular septum rich in conduction fibers.^25,26^

### Patients with ventricular pacing ≥20%

We examined patients with higher ventricular pacing burdens (≥20%) separately to evaluate its effect on cardiac function. Patients in the LBBAP group were more likely to receive ≥20% ventricular pacing (52% vs. 34%). This may be reflective of the operators’ avoidance of RVP (preference for BiVP) in patients likely to receive a high burden of ventricular pacing and stringent programming to avoid ventricular pacing in those receiving RVP as opposed to LBBAP. Both the groups were similar in baseline characteristics and echocardiographic variables, although the intrinsic conducted QRS was shorter in the LBBAP group as compared to the RVP group. This baseline difference is again likely due to the perceived safety with LBBAP, which permits operators to pace more liberally even in cases without atrioventricular blocks. Additionally, among the ≥20% ventricular paced population, the median ventricular pacing burden at 3 months was greater in the LBBAP group compared to the RVP group (98.8% vs. 90.0%). In line with previous studies, the paced QRS duration was shorter in LBBAP than in RVP.^25^

Among the subgroup with ≥20% ventricular pacing, the LBBAP patients had a favorable echocardiographic response, with the average LVEF improving and the average LVIDd decreasing as compared to vice versa in the RVP group. However, it is worth noting that the RVP controls for this subgroup analysis had a longer baseline QRS duration and thus may have been predisposed to developing cardiomyopathy. These findings can be interpreted to suggest that a wider baseline QRS duration may portend a greater chance of systolic cardiomyopathy and potential need for cardiac resynchronization therapy, and therefore an even stronger reason to consider LBBAP over RVP.

### Overall follow-up with ventricular pacing ≥20%

During the entire duration of follow-up, the LBBAP group had lower all-cause mortality, fewer HF hospitalizations, and fewer days hospitalized for HF; however, this difference is attributed to the large difference in the duration of follow-up between the two groups. Indeed, there were no statistical differences in the hazard rates for mortality or HF in the proportional hazards models that account for the differences in follow-up duration.

### Limitations

Our analysis is subject to several limitations. First, all typical limitations of a retrospective study apply, and our results may be biased by systematically missing data, especially missing outcomes of ECG and electrical parameters in the RVP group. Second, the procedures were conducted by seven operators without standardized protocols or endpoints. Third, the use of a historical cohort with different durations of follow-up affected the analysis of long-term outcomes. Finally, the imbalances in sex and device distribution between the two groups could have affected outcomes and electrical parameter data.

## Conclusions

LBBAP is increasingly recognized as a viable alternative to RVP. Key findings include a lower paced QRS duration, lower pacing impedance, and higher sensing in the LBBAP group, despite the procedure’s increased complexity and longer implant times. Importantly, LBBAP did not result in any higher rates of device-related adverse events, including lead dislodgements. Our analysis confirms the benefits of LBBAP, and we did not observe any adverse consequences from a practice-wide shift from RVP to LBBAP.
